# Supplementary material for: Cutibacterium spp. Infections after Instrumented Spine Surgery Have a Good Prognosis Regardless of Rifampin Use: A Cross-Sectional Study
Source: Antibiotics (Basel). 2023 Mar 4;12(3):518. doi: 10.3390/antibiotics12030518 (PMC10044575; doi:10.3390/antibiotics12030518)
Supplement: Supplementary file 1 [file antibiotics-12-00518-s001.zip › antibiotics-2231750-supplementary.pdf]

Supplementary Material:

Baseline Demographics (Monomicrobial only)			
	non-*Cutibacterium* N=260	*Cutibacterium* spp. N=16	p-value
**Age**	**57.15 (17.9)**	**37.15 (17.9)**	**0.0001**
**Median age and IQR**	**61.1 (46.9-71.0)**	**28.16 (18.5-53.3)**	**0.035**
BMI	28.05 (6.04)	25.6 (4.6)	0.195
Gender (female)	134 (51.5%)	9 (56.25%)	0.892
Corticoid treatment	23 (6.0%)	0 (0%)	0.38
Previous surgery	23 (7.3%)	4 (4.2%)	0.349
Charlson Comorbidity Index> 0	191 (50.8%)	21 (77.8%)	0.007
ASA=>2	352 (94.9%)	18 (66.7%)	0.001
Surgical Characteristics			
	non-*Cutibacterium* N=384	*Cutibacterium* spp. N=27	p-value
Emergency vs elective	19 (7.3%)	2 (9.5%)	0.396
**Fusion > 6 segments**	**38 (15.7%)**	**7 (46.7%)**	**0.002**
**Number of fused segments**	**3.82 (3.2)**	**6.07 (3.5)**	**0.011**
Surgical time	233.9 (126.1)	235.6 (151.4)	0.913
Cervical	22 (8.5%)	2 (13.3%)	0.516
Thoracic	88 (33.8%)	8 (53.3%)	0.124
Lumbar	217 (83.5%)	11 (73.3%)	0.311

Table S1: Baseline Demographics and Surgical Characteristics of the Monomicrobial Infection Subgroup. Numerical variables are expressed with mean values and standard deviations and categorical variables with number of cases and percentages. IQR= Interquartile Range, ASA= American Society of Anaesthesiologists.

Infection Characteristics (Monomicrobial only)			
	non-*Cutibacterium* N=260	*Cutibacterium* spp. N=16	p-value
**Mean time from surgery to infection (days)**	**156.3 (712.4)**	**827.3 (1642.8)**	0.027
**Median time from surgery to infection (days)**	**14 (7-24.7)**	**35 (10-1310)**	0.102
Infection within the first year	242 (93.1%)	11 (73.3%)	0.006
Infection within the first 90-days	229 (88.1%)	10 (66.7%)	0.017
**C reactive protein (mg/L)**	107.8 (110.6)	73.9 (100.6)	0.235
Erythrocyte sedimentation rate (mm/h)	67.1 (33.1)	73.0 (30.9)	0.65
Leucocytes (mm3)	108056.8 (4916.6)	9801.4 (3200.6)	0.429
Wound dehiscence	110 (42.3%)	6 (40.0%)	0.86
**Wound drainage**	**186 (72.1%)**	**7 (50%)**	0.076
Sinus tract	21 (8.1%)	1 (6.7%)	0.79
Fever>38ºC	118 (45.9%)	3 (21.4%)	0.073
Erythema, swelling	109 (42.4%)	3 (20%)	0.086
More than one debridement needed	24 (9.3%)	0 (0.0%)	0.217
Outcome (absence of treatment failure)	23 (8.9%)	0 (0%)	0.228
**Implant removal**	42 (16.2%)	5 (33.3%)	0.086

Table S2: Infection Characteristics of the Monomicrobial Infection Subgroup. Numerical variables are expressed with mean values and standard deviations (or as median and interquartile range (IQR) when indicated). Categorical variables are expressed with number of cases and percentages.
